# Third reported case of rare necrolytic migratory erythema associated with bacteraemia due to severe zinc deficiency after revisional Roux-En-Y gastric bypass: case report and literature review

**DOI:** 10.1007/s40519-021-01154-z

**Published:** 2021-06-01

**Authors:** Yassmin Salaheldin, Walid El Ansari, Esraa Aljaloudi, Wahiba Elhag

**Affiliations:** 1 Department of Bariatric Surgery/Medicine, Hamad General Hospital, Hamad Medical Corporation, Doha, State of Qatar; 2 Department of Surgery, Hamad General Hospital, Hamad Medical, Doha, State of Qatar; 3 College of Medicine, Qatar University, Doha, State of Qatar; 4 School of Health and Education, University of Skövde, Skövde, Sweden; 5 Department of Family Medicine, Hamad General Hospital, Hamad Medical Corporation, Doha, State of Qatar

**Keywords:** Necrolytic migratory erythema, Zinc deficiency, Revisional Roux-En-Y gastric bypass, Bacteraemia

## Abstract

**Introduction:**

Obesity is a risk factor for zinc deficiency. After bariatric surgery, non-compliance to diet/vitamin supplements, surgical complications leading to vomiting/diarrhea, poor follow-up and malabsorption can precipitate or exacerbate pre-existing zinc deficiency.

**Case report:**

We report a patient with rare necrolytic migratory erythema associated with bacteraemia due to severe zinc deficiency after revisional Roux-en-Y gastric bypass (following primary laparoscopic sleeve gastrectomy).

**Conclusion:**

Bariatric teams should screen patients before bariatric surgery for nutritional deficiencies and continue surveillance of their nutritional status after surgery. They should maintain a high index of suspicion for zinc deficiency in patients with skin rash after bariatric surgery.

**Level of evidence:**

Level V, case report.

## Introduction

Necrolytic migratory erythema (NME) is the most common cutaneous manifestation of glucagon-producing pancreatic neuro-endocrine tumors [1]. NME is also observed with chronic liver, kidney, and inflammatory diseases. It is also associated with intestinal malabsorption and nutritional deficiencies of essential amino acids, fatty acids and minerals such as zinc [2]. This form of NME is referred to as acquired acrodermatitis enteropathica [2, 3]. NME presents with demarcated erythematous patches, extensive crusting, scaling and discharge, centrally distributed involving the face, trunk and perineum [4], with stomatitis, cheilitis and alopecia [5].

The third highest zinc concentration is in the skin, crucial for its integrity, differentiation and proliferation of keratinocytes, anti-inflammatory effects, and wound healing [5]. Zinc is absorbed in the duodenum and jejunum [6]. Acquired zinc deficiency due to intestinal malabsorption is linked to NME, alopecia, alopecia areata, atopic dermatitis, and cutaneous ulcers [7].

Both obesity and bariatric surgery (BS) can cause zinc deficiency, probably due to consumption of calorie-dense nutritionally-poor foods [8]. Fasting zinc concentration was inversely related to body mass index (BMI) [9]; and obese individuals had lower zinc than lean controls [9]. Moreover, 50% of bariatric patients had zinc deficiency pre-surgery [10]. In terms of primary BS, the prevalence of zinc deficiency post-LSG, RYGB and biliopancreatic diversion/duodenal switch (BPD/DS) was 11–14%, 15–21% and 45–91%, respectively [10]. It remains unclear whether revisional RYGB is associated with more micronutrient deficiency compared to primary RYGB. We report a rare post-revisional RYGB NME due to severe zinc deficiency complicated by cellulitis and bacteremia. To the best of our knowledge, this is the third reported case of NME precipitated by severe zinc deficiency after revisional BS.

## Case presentation

**Fig. 1:**
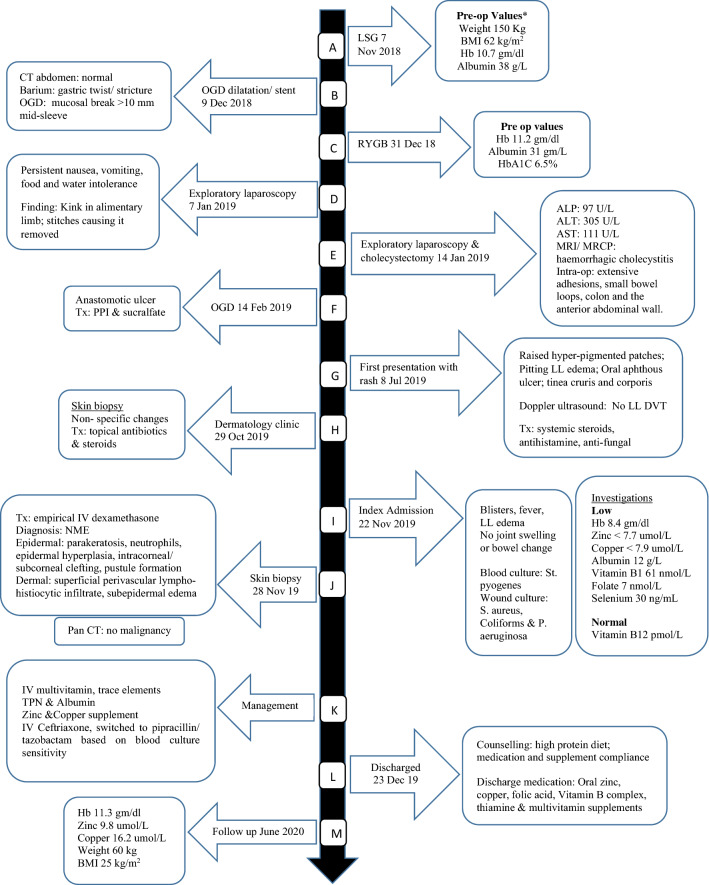
Timeline and sequence of events over 2 years. *Latest laboratory available on June 11, 2018; *LSG* laparoscopic sleeve gastrectomy, *Pre- op* Pre-operative, *BMI* body mass index, *Hb* Hemoglobin, *OGD* esophagogastroduodenoscopy, *CT* computed tomography, *RYGB* Roux-en-Y gastric bypass, *ALP* alkaline phosphatase, *ALT* alanine aminotransferase, *AST* aspartate aminotransferase, *MRI* magnetic resonance imaging, *MRCP* magnetic resonance cholangiopancreatography, *Tx* treatment, *PPI* proton pump inhibitor, *LL* lower limbs, *DVT* deep venous thrombosis, *St* Streptococcus, *S* Staphylococcus, *P* Pseudomonas, *IV* intravenous, *NME* Necrolytic migratory erythema, *TPN* total parenteral nutrition

We first outline the history, previous procedures and admissions preceding the index admission. Figure 1 depicts the sequence of events over 2 years.

A 28-year-old female presented to our emergency department (ER, Hamad General Hospital, largest tertiary care institution in Qatar) on November 22, 2019, with skin blisters, lower limb edema, pain and fever (Fig. 1i, index admission).

The patient had history of obesity (weight 150 kg, BMI 62 kg/m^2^) and had undergone LSG overseas in November 2018 (Fig. 1a). Three weeks later, she presented to our ER with nausea, vomiting and abdominal pain. Initial workup showed gastric twist/structure, and a mid-sleeve mucosal break (Fig. 1b). She underwent endoscopic dilatation with stent insertion (Fig. 1b), but with subsequent food and water intolerance, she required saving revisional RYGB (Fig. 1c). During her hospital stay, she was followed by multidisciplinary team of bariatric surgeon, physicians, physiotherapist and dietician. She was stable after the revisional surgery and was discharged. She was prescribed multivitamin supplementation (one tablet per day) containing vitamins A, B1, B6, B12, C, D, E, K, and thiamin, niacin, folate, biotin, pantothenic acid, calcium, iron, iodine, magnesium, zinc, selenium, copper, chromium, molybdenum, and sodium. Additional B complex tablets containing B1, B6, and B12 vitamins, as well as calcium carbonate daily and vitamin D weekly were prescribed. The patient was scheduled for follow-up for clinical evaluation and laboratory check.

She returned to the ER with recurrent nausea, vomiting and food intolerance. Her symptoms failed to improve with conservative management, so exploratory laparoscopy was decided and revealed an alimentary limb kink that was corrected surgically (Fig. 1d). The patient had persistent symptoms and elevated liver enzymes. Investigations (Fig. 1e) led to a second exploratory laparoscopy for cholecystectomy and release of intrabdominal adhesions (Fig. 1e). Her overall condition improved, she was discharged and was asymptomatic at follow-up a week later. A few months later, she developed anastomotic ulcer which was treated conservatively. Figure. 1f. She came for one follow-up appointment 2 weeks later at the bariatric surgery clinic but did not undertake the recommended blood test, and reported non-compliance to the multivitamin supplementation. She was advised to take the multivitamins regularly, and was given an appointment with the multidisciplinary team. However, she did not attend her follow-up appointments that were scheduled.

On July 8, 2019, she presented to the ER with a 3-day complaint of lower limb swelling and skin rash (Fig. 1g). She had no family history of skin or auto-immune disease. Ultrasound showed no deep venous thrombosis. She was admitted with provisional diagnosis of allergic reaction as she had consumed seafood. She was, treated and discharged after 1 week (Fig. 1g). Skin biopsy was later undertaken (Fig. 1h).

**Fig. 2:**
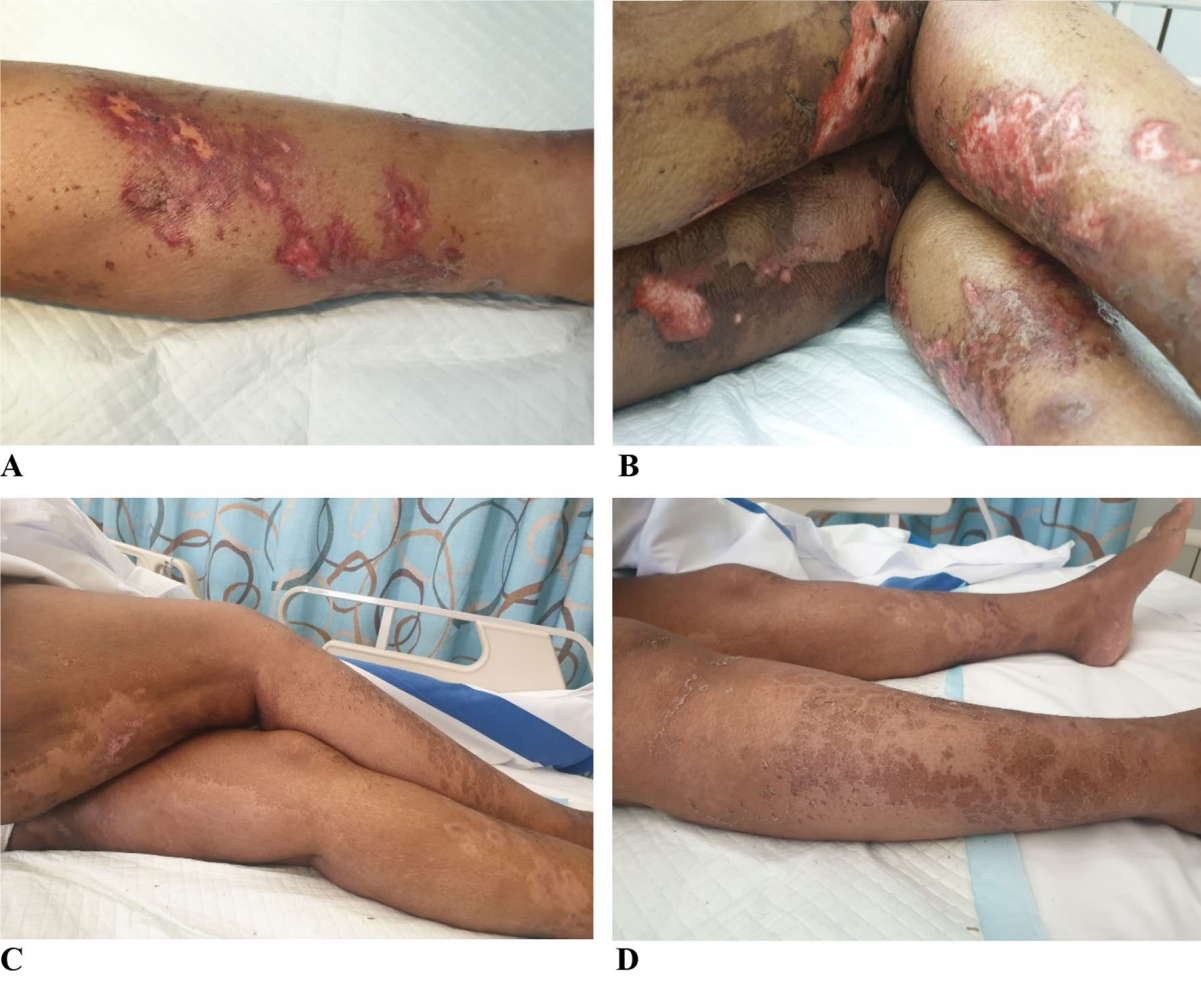
Lower limb NME skin manifestation at: time of admission (**a** left medial view, **b** dorsal view), and discharge (c, **d**)

**Fig. 3:**
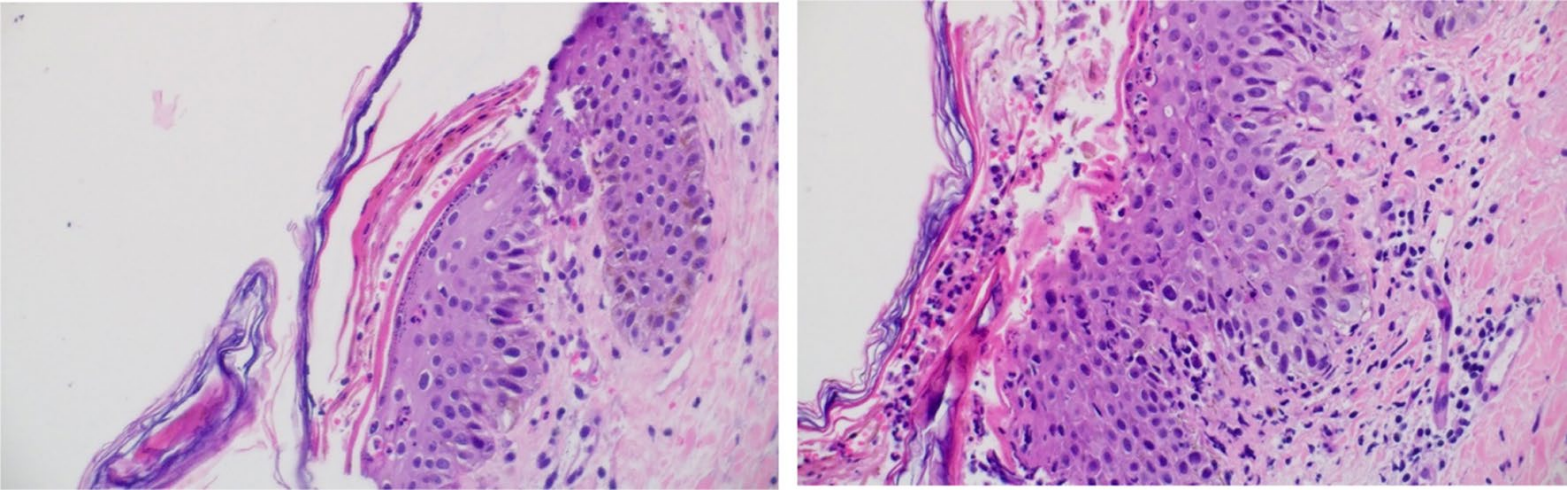
Parakeratosis with neutrophils, subcorneal clefting., inflammatory cell infiltrate

One month later, she presented again to the ER (index admission) with persistent symptoms and fever (Fig. 1i). She was conscious, oriented, looked ill and dehydrated. She had tender peri-oral ulcers, cheilitis, and generalized edema of the lower limbs with cellulitis of right leg and foot. She also had variable-sized blisters on the trunk and dorsa of the upper and lower limbs, some with foul-smelling discharge (Fig. 2a, b). Cardiovascular and chest examinations were within normal. Investigations were done (Fig. 1i), she was assessed by the dermatology team, started on empirical treatment (IV dexamethasone), but with minimal improvement. She underwent skin biopsy which suggested NME (Fig. 1j, Fig. 3), possibly due to abdominal neuro-endocrine tumor or severe zinc deficiency.

Pan CT ruled out occult malignancy (Fig. 1j). Zinc deficiency was confirmed by the bariatric team and treatment (Fig. 1k) lead to drastic improvement of the skin rash with almost complete resolution on some areas about 2 weeks later, with residual hyperpigmentation (Fig. 2c, d). She received IV zinc in total parenteral nutrition (TPN) 7 mg/day for 1 month duration. She was discharged in a stable condition (Fig. 1l). On the next follow up visit, her symptoms resolved and she had normal blood results (Fig. 1m).

## Discussion

We report severe zinc deficiency after revisional BS leading to rare cutaneous manifestations. Zinc deficiency with or without skin manifestations in morbidly obese individuals prior to BS has been reported [11]. Research found a 28% incidence of zinc deficiency in obese individuals prior to BS, probably due to consuming nutritionally inadequate meals rich in carbohydrates [12]. As the current patient had her initial LSG abroad, unfortunately, we are unable to speculate the zinc levels before her primary BS. Although her available history did not indicate skin lesions before her primary BS, such lack of skin lesions does not exclude zinc deficiency.

As for demographics, our patient, a 28-year-old female who underwent LSG followed by RYGB for surgical complications, agrees with the literature review we undertook (Table 1) where NME mostly afflicts middle aged patients and with a female predominance.

**Table 1:** Literature review of NME/acquired acrodermatitis enteropathica secondary to zinc deficiency after primary or revisional BS

	A	G	Skin lesion	DI	D	DD	Other RF	SI	Primary	Revisional	Taking MVT?	S Zin	S Alb	Diag	Histopathology	**DTx IV**	**TI**
Current Case 2020 Qatar	28	F	Ery, cr/sc	Ext., Abd., perineum, peri-oral	5 mo	Y	Stricture	Y	LSG	RYGB	N	< 7.7	low	HP	PK, Inf	1 mo	2 wk
Raghuna-than 2020 [18] USA	48	F	Ery, fissuring	Ext, Abd., Pn, Po	6 mo	Y	–	Y	RYGB	–	N	Low	Low	HP	PK, spongiosis	–	–
Giraldo-Villa 2019 [19] Colombia	46	F	Ery, fissuring, cr	Thighs, buttocks/Pn, feet	2 mo	Y	–	Y	RYGB	Intestinal resection/RYGB correction	–	4.43	–	HP	Confluent PK, EpH, lymp infiltrate	1 mo	–
Kurt 2019 [20] Turkey	40	F	Ery, ulcers, blisters Cheilitis	Ext, Po	3 mo	Y	Infectious gastroenteritis	–	LSG	–	–	7	Low	HP	Hyperkeratosis/PK eos inf	–	2 wk
Rana 2016 [21] USA	39	F	Ery, sc, fissures	Trunk, Ext, Po, periorbital	3 yrs	Y	–	–	RYGB	–	Y	2.9	Low	HP	PK, Inf Keratinocyte vaculation	1 wk	1 wk
Monshi 2015 [16] Austria	29	F	Ery, plaques/sc	Ext, genitoanal	–	–	Pregnancy/emesis	–	LSG	RYGB	–	5.8	Low	HP	Hyper/PK, neut inf	–	1 wk
Vick 2015 [22] USA	38	F	Ery, plaques, papules	Pn	8 mo	–	–	–	Gastric bypass	–	Y	7	–	HP	EpH/PK, dermal Inf	4 infusions	–
Jakubovic 2015 [23] Canada	34	F	Demarcated Ery, vesicles	Ext	4 mo	Y	–	–	RYGB	–	–	2	–	HP	Epidermal necrosis, Inf	–	–
Shahsavari 2014 [24] USA	39	M	Hyperpigmentation/dry sc	Ext	2 wk	N	Chronic alcoholism	Y	RYGB	–	–	4.12	–	HP	EpH, PK	–	–
Mankaney 2014 [25] USA	54	F	Ery, desq, excoriation	Lumbosacral, Pn, Inguinal, Ext	4 mo	–	Socioeconomic limits	–	RYGB	–	N	4.7	–	HP	PK, dermal Inf	–	4 wk
Bae-Harboe 2012 [3] USA	62	M	Ery, sc	palms/soles	4 wk	Y	–	–	RYGB	–	–	13.4	–	HP	EpH/PK	–	6 days
Cunha 2012 [17] Brazil	30	F	Ery, desq, sc	Generalized	7 mo	–	V/D, EF	–	SG	JB	Inadequate	5.2	Low	HP	Allergic reaction, vitamin def	2 mo	1 wk
Lewandowski 2007 [26] USA	43	F	Desq, sc, blisters, cheilitis, glositis	Ext, Torso	2 mo	Y	Nausea/D, Stricture, ulcer	Y	Distal RYGB	–	N	4.38	low	clinical	–	–	3–4 wk

Due to space considerations only the first author is cited

umol/L, – not reported, *A* Age, *Abd* abdomen, *Alb* albuminemia, *Cr* Crusting, *D* Duration of skin lesion, *DD* delayed diagnosis, *Def* deficiency, *Desq* desquamation, *DI* distribution of skin lesion, *Diag* Diagnosis, *DTx IV* duration of intravenous zinc supplementation, *EF* Enterocutaneous fistula, *Eos* eosinophilic, *EpH* epidermal hyperplasia, *ERY* Erythema, *Ext* extremities, *F* Female, *G* Gender, *HP* Histopathology, *Inf* inflammation/inflammatory cell infiltrate, *JB* jejunoileal bypass, *Lymp* lyphocytic, *M* Male, *Mo* months, *MVT* multivitamins, *N* No, *Neut* neutrophilic, *PK* Parakeratosis, *Pn* perineum, *Po* peri-oral, *S* serum *RF* risk factors, *RYGB* Roux-en-Y gastric bypass, *Sc* Scaling, *SG* Sleeve gastrectomy, *SI* Secondary Infection, *TI* time to improvement of symptoms, *V/D* Vomiting/Diarrhea, *Wk* weeks, *Y* Yes, *Yrs* years

Regardless of procedure type, deficiency is generally precipitated by non-compliance to diet and multivitamin supplements, inadequate intake, or surgical complications leading to vomiting or diarrhea [8]. As for procedure, LSG (primarily restrictive) can lead to or exacerbate zinc deficiency [13]. RYGB is a malabsorptive procedure that creates alterations in intestinal structure, bypassing a major part of the jejunum where zinc absorption takes place hence predisposing to micronutrients deficiency including zinc [8, 10]. While some authors found no statistically significant difference in risk of zinc deficiency between primary LSG and RYGB (34 % and 37 %, respectively) [14], others reported statistically lower zinc levels in primary RYGB compared to LSG [15].

It remains unclear, however, whether revisional BS is associated with worse nutritional outcomes compared to primary BS. Our patient underwent LSG, had multiple surgical complications, prolonged vomiting due to gastric twist, and was not compliant with the multivitamin supplements. As data are not available, we are unable to speculate her zinc levels in the period between her primary and revisional surgery; however, it is likely that she had zinc deficiency during this period due to the vomiting and inadequate oral intake. She then required revisional RYGB as a rescue procedure for her abdominal symptoms, which probably exacerbated her zinc deficiency. The literature on zinc levels after revisional BS is extremely sparse, as Table 1 shows that the majority of publications reported patients who underwent primary BS, with only two published cases of zinc deficiency post-revisional BS (in addition to the current case) [16, 17]. Future research could examine the risk of micronutrient and protein deficiency in revisional BS, particularly gastric bypass, compared to primary BS.

In terms of presentation, NME presents with erythematous raised plaques centrally distributed in areas of increased friction, forming blisters/flaccid bullae that crust and cause residual hyperpigmentation [2]. Complications with superimposed infections are common [2]. We are in agreement, as our patient had typical skin lesions complicated by cellulitis and bacteremia documented by blood culture. Others reported similar symptoms albeit with slight variability in the rash distribution (Table 1).

In terms of diagnosis, NME is initially commonly misdiagnosed, owing to its rarity and lack of exposure of general practitioners to this entity [2]. Skin biopsy is essential for diagnosis, showing parakeratosis and inflammatory cell infiltration [2]. Our patient had multiple visits to the ER and was initially diagnosed with urticaria, allergic reactions and tinea that did not improve with empirical treatment. She was eventually seen by a dermatologist and then diagnosed by skin biopsy, where histopathology showed keratinocyte necrotic changes and dermal infiltration with inflammatory cells suggestive of NME. All patients in Table 1 required skin biopsy for diagnosis and had typical histopathological findings similar to ours.

The management of NME relies on replacement of zinc and treatment of protein malnutrition [7]. The current case had near complete resolution of the rash 2–3 weeks after zinc supplementation in TPN, in agreement with other reported post-BS cases whose skin lesions significantly improved within 1–4 weeks of treatment with IV or oral zinc (Table 1). Prompt identification of the condition aids in early appropriate management and prevention of complications. Although this case study represents level V evidence, it is the third case study reported in the literature. It highlights that bariatric physicians should be aware of the skin manifestations associated with micronutrient deficiencies after BS.

## Conclusion

NME is a serious and rare dermatologic complication of severe zinc deficiency that is often misdiagnosed thus delaying treatment. The bariatric patient is at a twofold risk: pre-surgery, obesity is a risk factor for zinc deficiency; post-surgery, non-compliance to diet/vitamin supplements, surgical complications leading to vomiting/diarrhea, poor follow-up and malabsorption can precipitate or exacerbate pre-existing zinc deficiency. Patients should undergo nutritional screening before revisional BS, with prompt supplementation when deficiency is identified. Bariatric teams should have high index of suspicion for zinc deficiency in post-BS patients with skin rash. All patients need follow-up surveillance for nutritional status after BS. Future research could benefit from understanding the extent of micronutrient deficiency after revisional compared to primary BS.

### What is already known about this subject?

Zinc deficiency is common in patients with obesity and following bariatric surgery. It can present with skin symptoms, including alopecia, alopecia areata, atopic dermatitis, and cutaneous ulcers.

### What does this report add?

NME due to zinc deficiency post-bariatric surgery is misdiagnosed. Prompt identification/treatment is essential. This is the first case after revisional bariatric surgery reported from the Middle East.
